# Regulation of Renal Hemodynamics and Function by RGS2

**DOI:** 10.1371/journal.pone.0132594

**Published:** 2015-07-20

**Authors:** Patrick Osei-Owusu, Elizabeth A. Owens, Li Jie, Janaina S. Reis, Steven J. Forrester, Tatsuo Kawai, Satoru Eguchi, Harpreet Singh, Kendall J. Blumer

**Affiliations:** 1 Department of Pharmacology and Physiology, Drexel University College of Medicine, Philadelphia, Pennsylvania, 19102, United States of America; 2 Cardiovascular Research Center and Department of Physiology, Temple University, Philadelphia, Pennsylvania, 19140, United States of America; 3 Department of Cell Biology and Physiology, Washington University School of Medicine, St. Louis, Missouri, 63110, United States of America; The University of Tokyo, JAPAN

## Abstract

Regulator of G protein signaling 2 (RGS2) controls G protein coupled receptor (GPCR) signaling by acting as a GTPase-activating protein for heterotrimeric G proteins. Certain *Rgs2* gene mutations have been linked to human hypertension. Renal RGS2 deficiency is sufficient to cause hypertension in mice; however, the pathological mechanisms are unknown. Here we determined how the loss of RGS2 affects renal function. We examined renal hemodynamics and tubular function by monitoring renal blood flow (RBF), glomerular filtration rate (GFR), epithelial sodium channel (ENaC) expression and localization, and pressure natriuresis in wild type (WT) and RGS2 null (RGS2-/-) mice. Pressure natriuresis was determined by stepwise increases in renal perfusion pressure (RPP) and blood flow, or by systemic blockade of nitric oxide synthase with L-NG-Nitroarginine methyl ester (L-NAME). Baseline GFR was markedly decreased in RGS2-/- mice compared to WT controls (5.0 ± 0.8 vs. 2.5 ± 0.1 μl/min/g body weight, p<0.01). RBF was reduced (35.4 ± 3.6 vs. 29.1 ± 2.1 μl/min/g body weight, p=0.08) while renal vascular resistance (RVR; 2.1 ± 0.2 vs. 3.0 ± 0.2 mmHg/μl/min/g body weight, p<0.01) was elevated in RGS2-/- compared to WT mice. RGS2 deficiency caused decreased sensitivity and magnitude of changes in RVR and RBF after a step increase in RPP. The acute pressure–natriuresis curve was shifted rightward in RGS2-/- relative to WT mice. Sodium excretion rate following increased RPP by L-NAME was markedly decreased in RGS2-/- mice and accompanied by increased translocation of ENaC to the luminal wall. We conclude that RGS2 deficiency impairs renal function and autoregulation by increasing renal vascular resistance and reducing renal blood flow. These changes impair renal sodium handling by favoring sodium retention. The findings provide a new line of evidence for renal dysfunction as a primary cause of hypertension.

## Introduction

Essential hypertension is marked by functional changes in primary determinants that control arterial blood pressure, including augmented total peripheral vascular resistance and/or increased cardiac output[1, 2]. Increased peripheral resistance can result from defects in the production of and/or response to endothelium-derived vasodilator factors that counteract vasoconstriction by norepinephrine release from the sympathetic nervous system or vasoconstrictor substances including angiotensin II and vasopressin. Increased cardiac output can be caused by defects in water and electrolyte homeostasis in the kidney parenchyma, thereby leading to sodium and water retention and blood volume expansion. Thus the kidney plays a major role in long-term blood pressure control[3, 4].

Unlike many organs where blood flow can be regulated over a wide range to meet changes in metabolic demand, the kidney is unique in that blood flow is maintained within a narrow range to sustain glomerular filtration rate (GFR) and water and electrolyte reabsorption. Despite changes in systemic blood pressure, renal blood flow (RBF) is kept constant due to autoregulation at pre-glomerular afferent arterioles, which are the major site of renal vascular resistance regulation[5, 6], and to a lesser degree by autoregulation at post-glomerular efferent arterioles[7]. Myogenic constriction and tubuloglomerular feedback mechanisms mediate renal autoregulation; however, intrinsic factors including nitric oxide and agonists for guanine nucleotide-binding protein (G protein)-coupled receptors (GPCRs), particularly angiotensin II via the renin-angiotensin system, regulate RBF by modulating renal vascular resistance (RVR) in pre- and post-glomerular arterioles[8, 9]. Constant RBF ensures that GFR also remains unchanged within the renal autoregulation range, which is key to maintaining water and electrolyte homeostasis by the renal tubular system. Therefore, defects in renal hemodynamics that impair RBF and thus GFR potentially impact long-term blood pressure homeostasis by causing changes in water and electrolyte excretion and reabsorption. Water and electrolyte homeostasis is mediated by kidney-intrinsic mechanisms, which can be modulated by extrinsic factors, including the nervous, endocrine, and paracrine systems[10–12] via the activation and regulation of GPCR signaling.

Signaling by G proteins plays several important roles in blood pressure control. Many agonists that affect blood pressure activate receptors that signal via one or more members of the heterotrimeric G protein family (G_i/o_, G_q/11_, G_s_, and G_12/13_)[13, 14]. Aberrant G protein signaling contributes to the development and establishment of hypertensive disorders[14–16]. Accordingly, GPCRs and the signaling pathways they control remain predominant therapeutic targets of anti-hypertensive medications, including ANG II and beta-adrenergic receptor blockers.

GPCR signaling is controlled by a family of regulator of G protein signaling (RGS) proteins, which act as GTPase-activating proteins (GAPs) for G protein alpha subunits by accelerating the hydrolysis of GTP[17, 18]. By acting as GAPs, RGS proteins regulate the kinetics and amplitude of G protein signaling[19]. Among ~30 known mammalian RGS proteins[20], RGS2 is the most potent GAP towards G_q/11_-class G proteins[21, 22]. RGS2 deficiency causes hypertension in mice[15], and certain human hypertension populations harbor mutations that include *Rgs2* single nucleotide polymorphisms predicted to reduce expression or function[23–26]. RGS2 deficiency in mice is known to augment GPCR-induced vasoconstriction and impair endothelium-dependent vasodilation[27, 28], which are hallmarks of essential hypertension. RGS2 deficit has been shown to attenuate vasopressin-mediated regulation of water excretion[29]. Using kidney cross-transplantation, Gurley and colleagues previously showed that RGS2 deficiency in the kidney is sufficient to cause hypertension[30]. Transplantation of kidneys from RGS2-/- into wild type mice caused elevated ambulatory blood pressure. Conversely, hypertension in RGS2-/- mice was abolished following receipt of kidneys from wild type donors. While these findings clearly demonstrated the indispensable role for RGS2 in the kidney, how renal function is regulated by RGS2 remains unknown.

Accordingly, here we have assessed whether RGS2 deficiency affects GFR, RBF, and pressure natriuresis, which are crucial for blood pressure control by the kidney. Our study is the first to identify effects of RGS2 deficiency on renal function. Our results indicate that loss of RGS2 reduces RBF, GFR and sodium excretion. These findings suggest that hypertension resulting from downregulation or loss of RGS2 in humans may be attributable in part to aberrant renal vascular and tubular function.

## Methods

### Animals

Studies were performed in accordance with protocols approved by the Animal Studies Committees of Drexel University College of Medicine and Washington University School of Medicine. All experiments were performed at both institutions using 3–4 month-old male mice that have been backcrossed more than ten generations into the C57BL/6 background (Charles River). Generation of RGS2-/- mice has been described previously[31]. Mice were provided access to food and water ad libitum in our institution’s animal facility at 22°C and a 12-h light/dark cycle.

### Baseline glomerular filtration rate determination

To assess baseline renal function, we determined GFR in anesthetized mice. We used a modified procedure for measuring GFR in conscious mice based on plasma clearance of fluorescein isothiocyanate-inulin (FITC-inulin) following a bolus intravenous injection[32]. In this procedure, 5% FITC-inulin (Sigma-Aldrich, St. Louis, MO) was dissolved in 0.9% isotonic saline filtered with a 0.22 μm filter. Under 1.5% isoflurane anesthesia, each animal received a bolus injection of 3.74 μl/g body weight of 5% FITC-inulin solution via a venous catheter implanted in the right jugular vein. After FITC-inulin injection, arterial blood was sampled via a carotid catheter at 3, 7, 10, 15, 35, 55, and 75 min. At each blood sampling point, drawn blood (30 μl) was replaced with the same volume of heparinized saline. At the end of the experiment, the left kidney weight was determined following euthanasia by cervical dislocation under deep anesthesia. Blood samples were centrifuged for 5 min at 5000 rpm after which 10 μl of plasma was diluted with 40 μl of HEPES buffer (pH 7.4). FITC-inulin in each sample in a 96-well plate was measured with 485 nm excitation and 538 nm emission using Synergy HT multi-mode microplate reader (Biotek, Winooski, VT). GFR was calculated using a two-compartment clearance model as previously described[33].

### Glomerular number and surface area determination

Following GFR measurement by plasma clearance of FITC-inulin, the left kidney was accessed through a flank incision. The renal artery and vein were tied and the kidney excised. After decapsulation, the kidney was cut into small pieces and macerated in 30 ml of 50% (vol/vol) HCl solution in water at 37°C and continuously stirred for 90 min, as previously described[34]. After replacing the HCl solution with the same volume of distilled water, 1 ml of the kidney suspension was placed into each chamber of a four-chamber microscope slide. The number of glomeruli in each chamber was counted using a microscope with 10x magnification. The number of glomeruli per kidney was determined by taking the average of four counts per slide and multiplying by the total volume of the kidney suspension. To determine glomerular surface area, kidney sections and histological staining were performed following a previously described protocol[35]. Briefly, paraffin-embedded kidneys were cut into two equal halves in sagittal orientation, and four sections (two sections from each half, 5-μm thick) from each half were stained with eosin and hematoxylin. A Zeiss Axio Imager M2 equipped with Neurolucida image acquisition and analysis software was used to trace every superficial and cortico-medulllary glomerulus on each kidney section. Glomerular size was estimated from the measurement of surface area. Cross sectional area of glomeruli from sections at the same level of each genotype was measured. To control for differences in size of glomeruli from the cortical surface to the edge of the renal medulla, two groups of glomeruli in each section were compared: those close to the edge of the cortex and those close to the cortico-medullary line.

### Kidney fibrosis staining and quantification

Kidneys were fixed in formalin followed by tissue processing, mounting, and sectioning. To determine the presence of perivascular fibrosis, sections were stained in Sirius Red (EMS, Hatfield PA). Briefly, after paraffin removal and re-hydration, sections were stained in equal parts Weigert’s Iron Hematoxylin A and B (EMS, Hatfield PA) for 10 min at room temperature. Sections were then washed twice in distilled water for 3 min per wash. Sirius red was added for 1 h at room temperature. Slides were washed twice in 0.01N HCl for 3 min per wash. Sections were then dehydrated and penetrated using ethanol and xylene, respectively. Images were visualized on an Olympus IX81 inverted microscope using a Photometrics CoolSNAP HQ CCD camera and were acquired with SPOT 4.7 Basic software. Analysis was conducted using NIH ImageJ 1.49g software. To calculate perivascular fibrosis, the value of fibrosis area was subtracted from vessel area and divided by the true area of the vessel. True area was calculated by vessel outer perimeter^2^ divided by 4π. The value generated was the area of the vessel in true circular form. In total, 4 randomly selected samples per group were used for analysis. 3 representative renal vascular images were analyzed per sample.

### Extracellular fluid volume expansion and pharmacological challenge

Under isoflurane anesthesia, wild type and RGS2-/- mice were instrumented simultaneously with a renal flow probe (0.5PSB Nanoprobe with handle, Transonic) for RBF measurement, a fluid filled carotid artery catheter for blood pressure recording, a jugular vein catheter for fluid and drug infusion, and a bladder catheter for urine collection. Immediately after the surgical procedure was completed, the mice were given a bolus injection of isotonic saline (8 μl/g body weight) containing 2% bovine serum albumin and 0.33% FITC-inulin followed by continuous infusion of the same solution at 0.5 μl/min/g body weight. After 30 min of equilibration, baseline urine was collected for another 30 min, and blood sample was collected (50 μl) for the measurement of hematocrit, GFR and plasma electrolyte concentration. This was followed by acute administration of L-NAME (30 mg/kg, i.v.).

### Pressure natriuresis studies

Previously described methods for assessing pressure natriuresis in rodents developed by Roman, Cowley and others[36–38] were followed with minor modifications. Briefly, wild type and RGS2-/- mice were uninephrectomized at least two weeks before the experiment.

The right kidney was exteriorized and retracted via the retroperitoneal cavity, following a right flank incision under isoflurane anesthesia. Two pieces of 6–0 silk were tied around the right renal vascular pedicle and the ureter, after which the kidney was excised. The right adrenal gland was also removed after tying off all major blood vessels to the organ. The incision was closed by first suturing the muscle followed by closing of the skin incision with surgical staples and tissue glue.

On the day of the experiment, uninephrectomized mice were anesthetized with isoflurane and surgically instrumented with a fluid-filled carotid artery catheter for blood pressure measurement, a PE-10 jugular vein catheter for fluid and drug delivery, and a bladder catheter for urine collection. 6–0 silk ligatures were placed loosely around the celiac and the superior mesenteric arteries. The abdominal aorta below the left kidney was isolated from the vena cava and surrounding connective tissue to allow for stepwise increases in renal perfusion pressure by abdominal aortic occlusion. The left adrenal gland was then removed, and the kidney denervated by rubbing 10% phenol in 100% ethanol around the renal artery at the point of entry into the kidney. Shortly after surgical procedures were completed, mice were given a bolus injection of phosphate-buffered saline (PBS; 8 μl/g body weight) cocktail containing 2% bovine serum albumin, 1 μg/ml norepinephrine, 0.5 ng/ml arginine vasopressin, 0.2 mg/ml hydrocortisone, and 0.2 μg/ml aldosterone, followed by continuous infusion of the same solution at 0.5 μl/min/g body weight according to a previously described protocol[37]. Following a 45 min equilibration period, renal function was examined over three consecutive 20 min clearance periods: In period 1, baseline parameters and urine were collected; in period 2, arterial blood pressure and renal perfusion pressure were elevated by occluding the celiac and the superior mesenteric arteries with 6–0 silk ligatures; in period 3, renal perfusion pressure was further elevated by occluding the abdominal aorta below the left kidney with a metal clamp. At the midpoint of each clearance period, an arterial blood sample (100 μl) was drawn for determination of electrolyte concentration. Heparinized donor blood (100 μl) from RGS2+/- littermates was then injected to replace the lost volume after each blood draw. Kidney weight was determined at the end of the experiment. Plasma and urine electrolyte analyses were performed by Washington University School of Medicine’s Core Laboratory for Clinical Studies. Hemodynamic data were recorded and analyzed with PowerLab system (ADInstruments).

### Tissue preparation for immunohistochemistry

At the end of each volume expansion experiment, the mice were deeply anesthetized with ketamine/xylazine (ketamine; 43 mg/kg, i.p., and xylazine; 6 mg/kg, i.p.) and perfused through the left ventricle with 4% sucrose in 4% paraformaldehyde-phosphate buffered saline solution. The fixed kidneys were immersed in 100 mMTris-HCl solution for 30 min and transferred into 30% sucrose in PBS overnight. After embedding in TissueTek OCT compound in a Cryomold vinyl mold, 30 μm-thick sections were cut and immediately used for immunofluorescence labeling. To this end, kidney sections were incubated in 0.01% Triton X-100 in TBS for 10 min followed by 1 hr incubation in 1% non-fat milk in TBS-Tween and overnight incubation with αENaC (1:500, Santa Cruz) or NHE-3 (1:500, Santa Cruz) primary antibody at 4°C. Following several washes, the tissue was incubated with Alexa Fluor (anti-rabbit IgG, Alexa Fluor 488 or 568 conjugate) secondary antibody for 1 h at room temperature, then washed several times and mounted on slides. Images were obtained using Olympus IX81 laser scanning confocal microscope. All images were acquired using a 60x oil immersion objective (NA = 1.42) at a pixel size of 103 nm. To quantify αENaC translocation and clustering, we analyzed αENaC puncta sizes in raw images acquired by a confocal microscope as previously described[39]. We determined the sizes of ENaC punctae by measuring the half-maximum of full width using pixel intensity peaks as a function of distance. On an average, 10 individual puncta were measured from every image. We then generated puncta size frequency distribution plots to determine the distribution of ENaC clusters along the lumen of renal tubules in each genotype. Frequency distributions were fitted to multiple Gaussian functions to determine the size of the most predominant ENaC clusters in each genotype.

### Renal autoregulation

Mice were instrumented with carotid artery and jugular vein catheters as described above, except that uninephrectomy was not performed. For acute renal blood flow (RBF) measurements, a renal flow probe (0.5PSB Nanoprobe with handle, Transonic) was placed around the left renal artery following a left flank incision. 6–0 silk ligatures were placed loosely around the celiac and the superior mesenteric arteries. At the end of the surgery, the mice were given a bolus injection of 2% BSA/saline (8 μl/g body weight), followed by a continuous infusion of the same solution at 0.5 μl/min/g body weight while blood pressure and RBF were recorded continuously at 1000 Hz. After 30 min of equilibration, renal perfusion was increased by tightening the ligature around the celiac and mesenteric arteries while blood pressure and RBF were continuously recorded for 20 min.

### Data analysis

Hemodynamic data were processed and analyzed as previously described[40, 41]. The recorded blood pressure and RBF data were analyzed offline using SigmaPlot 12.5 software. The 1000-Hz blood pressure and RBF data were resampled and smoothed by a running average over 100 point segments. RVR was calculated as mean arterial pressure (MAP) divided by RBF and normalized to body weight. Renal conductance was calculated as RBF divided by MAP and normalized to body weight.

The speed of the myogenic response was assessed by determining the slope of a trendline drawn for the first 10 points of the rapid rising phase of RVR, following a step increase in MAP by occlusion of the celiac and superior mesenteric arteries. Steady-state autoregulation at 2 minutes following the step increase in MAP was determined by calculating the autoregulatory index (AI) using the following equation:

$$\text{AI}=\left[\frac{\text{RBF}2-\text{RBF}1}{\text{RBF}1}\right]/\left[\frac{\text{MAP}2-\text{MAP}1}{\text{MAP}1}\right]$$

Where RBF1 and MAP1 are the average baseline values taken at 10 s before the step increase in pressure, and RBF2 and MAP2 are the average of values taken from 110 s to 120 s after the step increase in pressure.

### Statistical analysis

All data are mean ± standard error. Baseline GFR was analyzed by one-way analysis of variance (ANOVA). Unpaired student’s t test was used to compare glomerular number and surface area. For assessment of within-group effects of increasing perfusion pressure on urine output, sodium excretion, and renal hemodynamics, one-way ANOVA with repeated measures was used, and two-way ANOVA with repeated measures was used for between-group comparisons with Neuman-Keuls post-hoc tests. All other comparisons were performed using student’s t test or ANOVA where appropriate. A *p*<0.05 was considered statistically significant.

## Results

### Decreased baseline GFR in RGS2-/- mice

**Fig 1:**
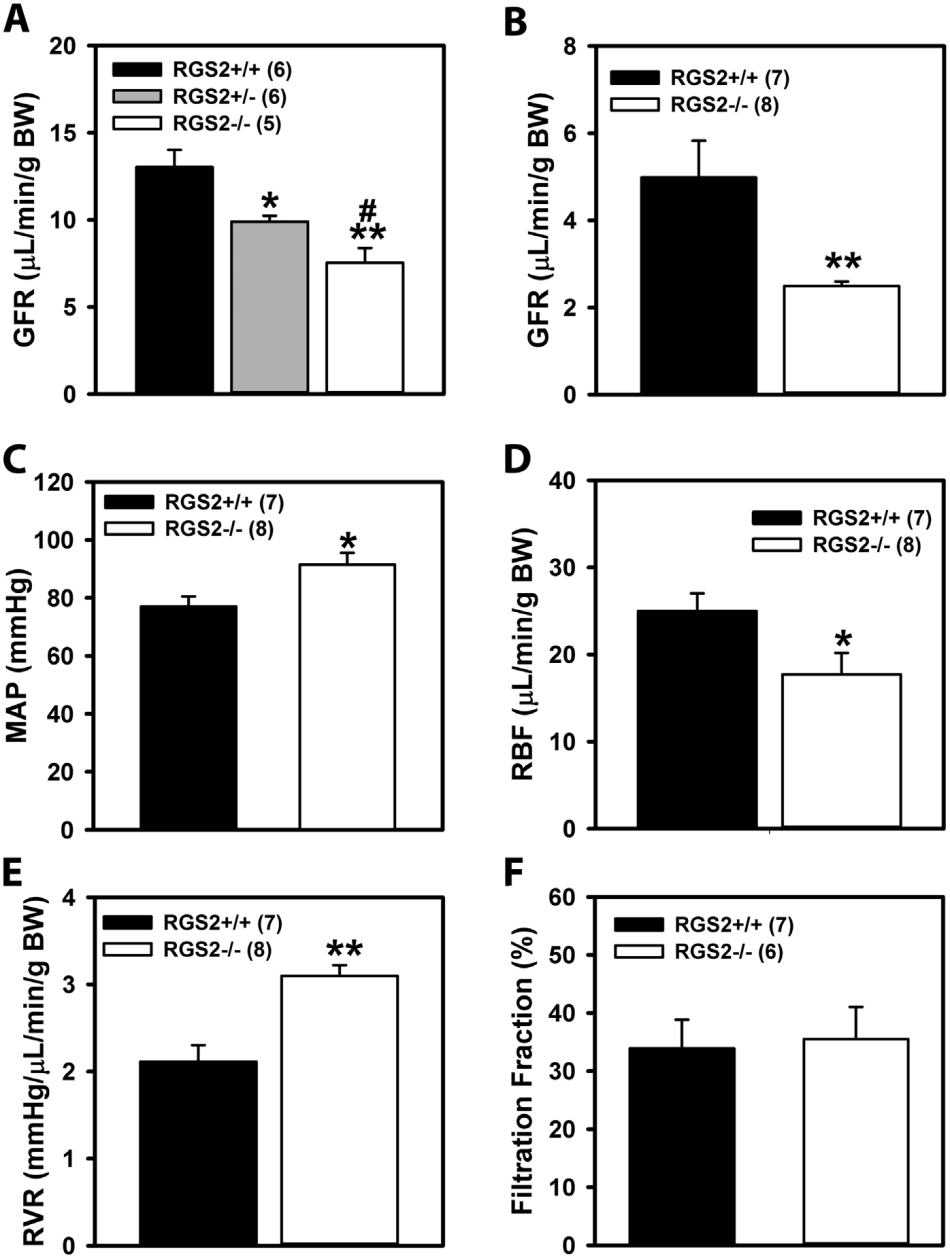
Baseline glomerular filtration rate (A, B), mean arterial pressure (MAP; C), renal blood flow (RBF; D), renal vascular resistance (RVR; E), and filtration fraction (F) of wild type (RGS2+/+), RGS2 heterozygous (RGS2+/-), and RGS2 knockout (RGS2-/-) mice. Values are mean ± SE. *^,^**P < 0.05, 0.01 vs. RGS2+/+; ^#^ *P* < 0.05 vs. RGS2+/-. BW, body weight.

Gurley and colleagues showed previously that RGS2 deficiency in the kidney plays a causal role in blood pressure elevation in RGS2-/- mice[30]. To determine how the loss of RGS2 affects renal function, we analyzed GFR, glomerular number and size, renal hemodynamics, and pressure natriuresis in wild type and RGS2-/- mice. First, we determined baseline GFR in anesthetized mice by measuring plasma clearance of FITC-inulin following a single bolus intravenous injection. GFR was markedly decreased in both RGS2-/- and RGS2+/- mice compared to congenic wild type controls (Fig 1A). We confirmed that GFR was markedly reduced in RGS2-/- mice by using urine clearance of inulin with continuous infusion of saline/BSA containing FITC-inulin. As shown in Fig 1B, this approach verified that RGS2-/- GFR was reduced relative to wild type mice. We noted that GFR measured by plasma inulin clearance was approximately two times higher than that measured by urine inulin clearance, as previously reported[42]. This difference could have resulted from the GFR estimation calculation used in the former method[32]. Nevertheless, both methods showed a similar trend of decreased GFR in RGS2-/- mice. We next determined whether decreased GFR in RGS2-/- mice might be due to reduction in nephron number. The average number of nephrons per kidney was determined by counting the number of isolated glomeruli per kidney after hydrochloric acid maceration. As shown in Table 1, nephron number was higher in RGS2-/- mice compared to wild type controls (RGS2-/-; 12270 ± 677 vs. WT; 9209 ± 421, p<0.01). Because wild type and RGS2-/- kidney weights were similar, we determined whether there was any change in the size of glomeruli that potentially compensates for differences in glomerular number. As shown in Table 1, the average surface area of superficial glomeruli was smaller in RGS2-/- relative to wild type controls. Cortico-medullary glomeruli showed a similar trend, though the difference did not reach statistical significance (p = 0.06). These data indicated that impaired GFR is caused by defective kidney function but not glomerular deficit.

**Table 1:** Kidney morphometry of 3-4-month old wild type (RGS2+/+) and RGS2 knockout (RGS2-/-) mice. Values are mean ± SE.

Genotype	RGS2+/+ (n = 7)	RGS2-/ (n = 5)	P value
Body weight (g)	25 ± 1	29 ± 1	0.047
Kidney weight (g)	0.179 ± 0.01	0.198 ± 0.02	0.326
Number of glomeruli per kidney	9209 ± 421	12270 ± 677	0.002
Superficial glomerular surface area (μm^2^)	2749 ± 57	2423 ± 104	0.012
Cortico-medullary glomerular surface area (μm^2^)	5766 ± 442	4801 ± 279	0.060

### Decreased total RBF and augmented baseline RVR in RGS2-/- mice

Previous *in vitro* studies have shown that RGS2 modulates stretch- and GPCR-induced vasoconstriction of renal interlobar arteries[27]. Here, we determined whether reduced GFR observed in RGS2-/- mice might be due to decreased renal perfusion arising from increased vasoconstriction. Fig 1C–1E summarize data including baseline mean arterial pressure (MAP), RBF, and RVR of wild type and RGS2-/- mice under isoflurane anesthesia. Baseline MAP was ~10 mmHg greater while RVR also increased by ~1 mmHg/μl/min/g body weight in RGS2-/- mice compared to wild type controls. These changes were accompanied by a reduction in baseline RBF (Fig 1D) while filtration fraction was unchanged (Fig 1F) in RGS2-/- mice. These results suggested that decreased GFR in RGS2-/- mice is due at least partly to decreased renal perfusion and increased resistance of the renal vasculature.

### Increased renal perivascular fibrosis in RGS2-/- mice

**Fig 2:**
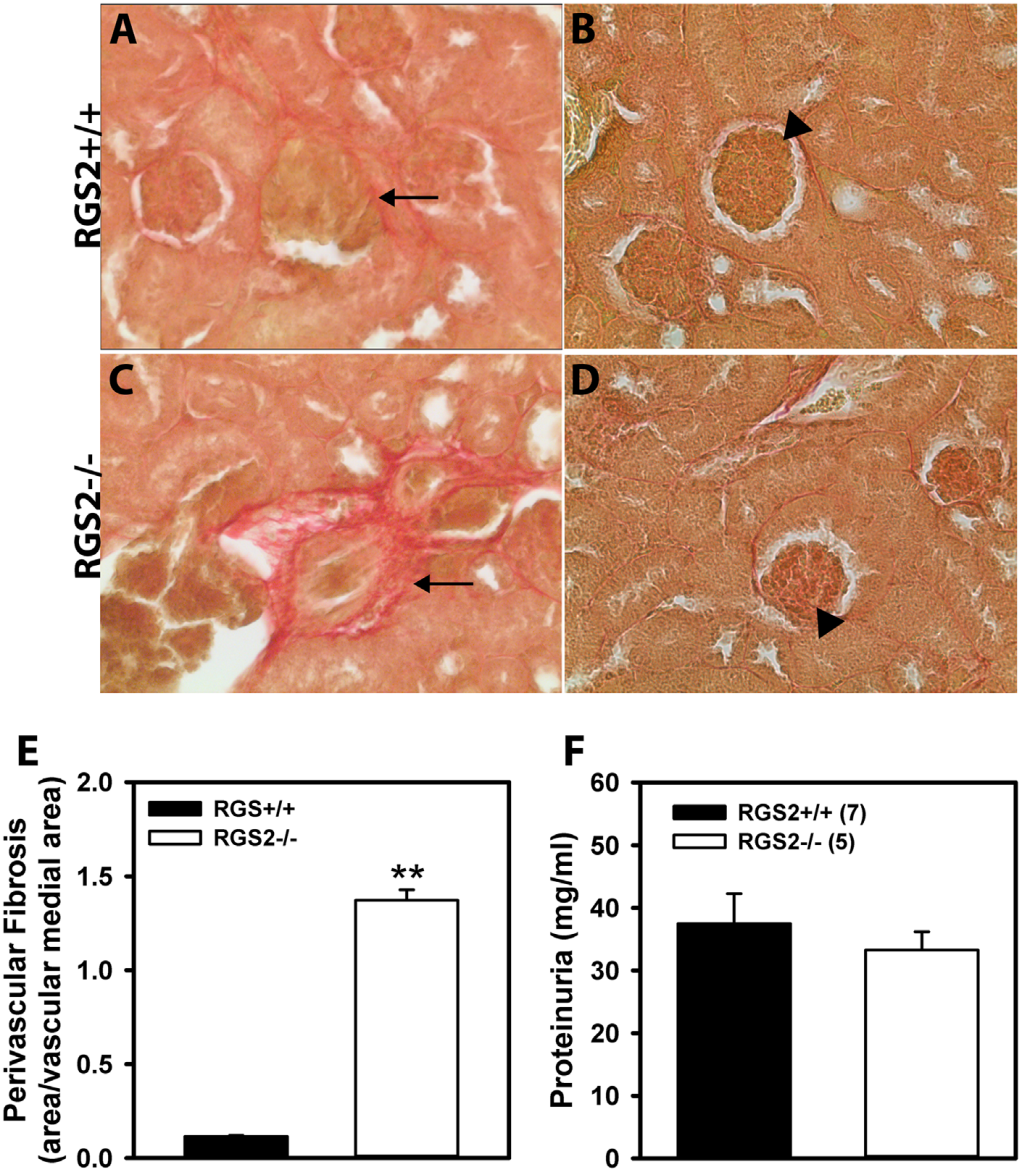
Structural analysis of renal vasculature in wild type (RGS2+/+) and RGS2 knockout (RGS2-/-) mice. Sirius red staining for tissue fibrosis in RGS2+/+ (A and B) and RGS2-/- (C and D) kidneys. Arrows indicate renal microvessels and arrowheads indicate glomeruli. E, Quantification of perivascular fibrosis in RGS2+/+ and RGS2-/- kidneys. RGS2-/- renal microvessels showed a 11-fold increase in perivascular fibrosis relative to RGS2+/+ vessels. F, 24-hr proteinuria was similar between RGS2+/+ (n = 7) and RGS2-/- (n = 5) mice. **P < 0.01 vs. RGS2+/+.

RGS2 deficiency results in increased vasoconstrictor signaling[27] in renal arteries and acceleration of renal fibrosis induced by ureteral obstruction[43]. Because chronic vasoconstriction can lead to tissue hypoperfusion and ischemic damage, we determined whether decreased RBF in RGS2-/- resulted in renal injury. As shown in Fig 2A and 2C, RGS2-/- mice developed remarkable perivascular fibrosis that was absent in wild type controls. When normalized to total vessel cross-section, RGS2-/- kidneys showed a 11-fold increase in perivascular fibrosis relative to wild type (Fig 2E). In contrast to other models of renal fibrosis, we did not observe any significant glomerular or interstitial fibrosis (Fig 2B and 2D). Furthermore, urinary protein excretion was unaffected in RGS2-/- mice (Fig 2F), suggesting the absence of glomerular filtration unit damage.

### Effect of RGS2 deficiency on whole kidney steady-state autoregulation

**Fig 3:**
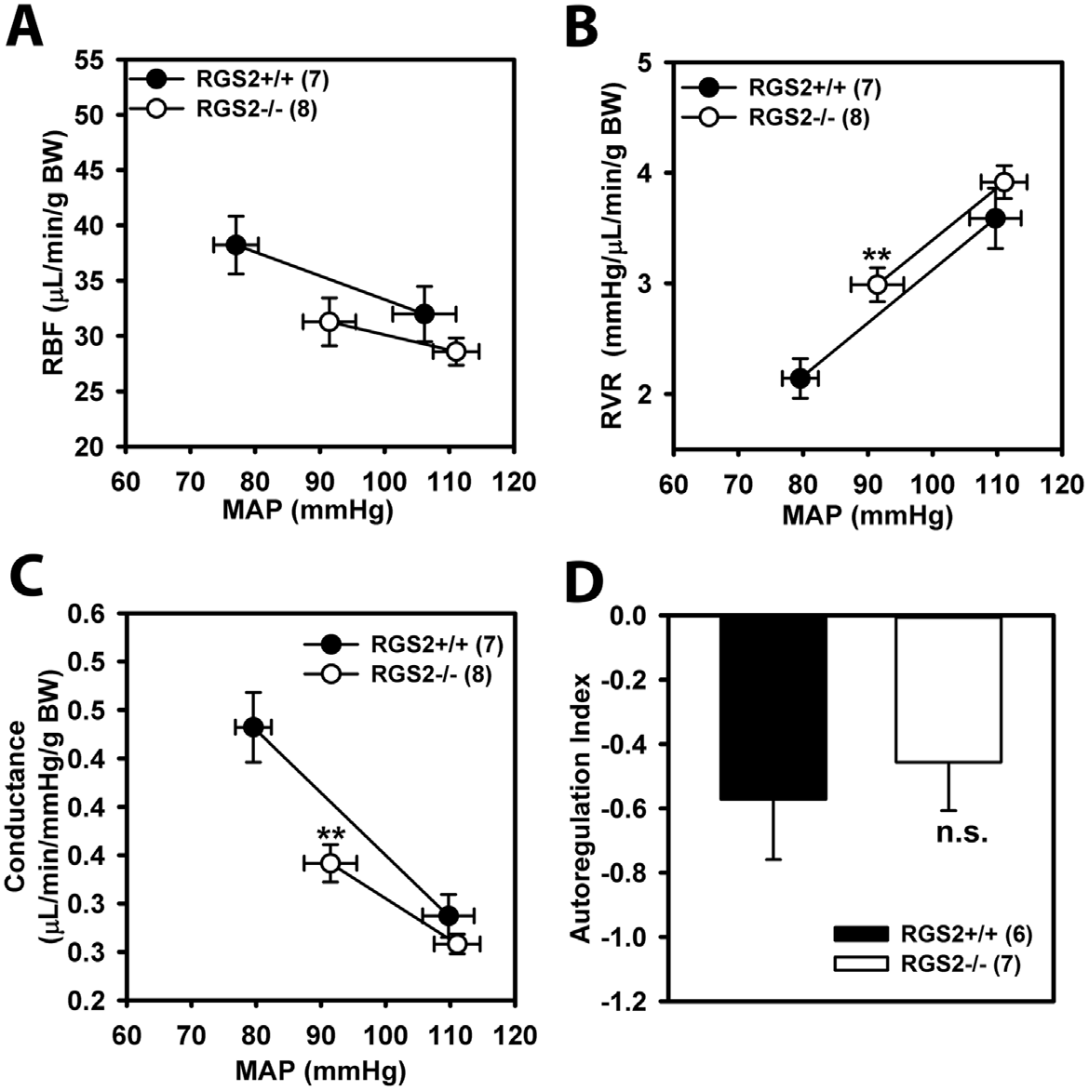
Effects of increasing renal perfusion pressure (MAP) on renal blood flow (RBF, A), renal vascular resistance (RVR, B), and renal conductance in wild type (RGS2+/+, n = 7) and RGS2 knockout (RGS2-/-, n = 8) mice. Increased MAP caused a greater change in RVR and conductance of RGS2+/+ relative to RGS2-/- mice. D, autoregulation index indicating no difference in steady-state autoregulation efficiency between RGS2+/+ and RGS2-/- mice. Values are mean ± SE. **P < 0.01 vs. RGS2+/+ baseline RVR and conductance; n.s., not significant vs. RGS2+/+. BW, body weight.

To determine how baseline RBF and GFR were decreased in RGS2-/- mice, we assessed steady-state autoregulation under isoflurane anesthesia. Fig 3 shows MAP-RBF, MAP-RVR and MAP-conductance relationships before and 2 min after a step increase in renal perfusion pressure. Steady state MAP increased ~30 mmHg above baseline in wild type whereas it increased ~20 mmHg above baseline in RGS2-/- mice. A similar shift in RBF, RVR and conductance was observed in wild type and RGS2-/- mice (Fig 3A, 3B and 3C). However, the magnitude of change in RVR (WT: 1.6 ± 0.3 vs. RGS2-/-: 1.0 ± 0.1 mmHg/μl/min/g body weight) and conductance (WT: -0.18 ± 0.04 vs. RGS2-/-: -0.1 ± 0.02 μl/min/mmHg/g body weight) was greater in wild type relative to RGS2-/- mice. As shown in Fig 3D, autoregulatory index (AI), a measure of autoregulation efficiency was not different between the groups. In addition, no correlation was found between baseline MAP and AI in both genotypes (data not shown). Together the results indicate that RGS2 deficiency decreases the responsiveness but not the steady-state autoregulation of renal conductance.

### RGS2 deficiency decreases the kinetics of RBF, RVR, and renal conductance response to a step increase in renal perfusion pressure

**Fig 4:**
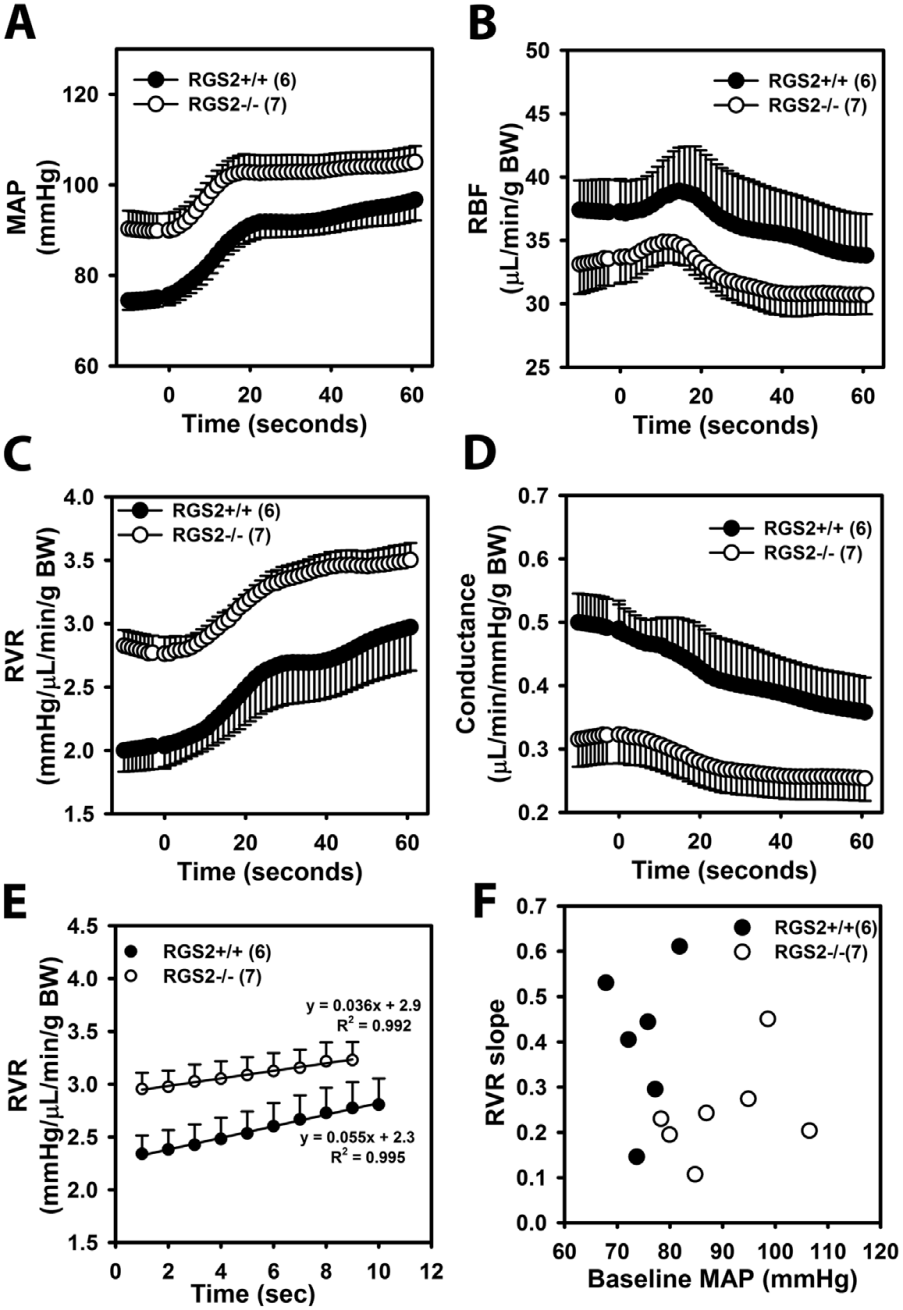
Time course of renal hemodynamic response to a step increase in perfusion pressure. Fig A-D show responses of mean arterial pressure (MAP, A), renal blood flow (RBF, B), renal vascular resistance (RVR, C), and renal conductance (D), 10 seconds before and 60 seconds after occluding celiac and superior mesenteric arteries of isoflurane-anesthetized mice. E, a trendline drawn through the first 10 seconds after the beginning of increase in RVR following a step increase in MAP to determine the speed of myogenic response. Each point is the average from several animals (RGS2+/+; n = 6 and RGS2-/-; n = 7) at the time points in each group. The average slope of each trendline shown in the linear equations in E is equal to the mean of slopes from trendlines of all animals (used in panel 3F) in each group. F, scatter plot showing the correlation between baseline MAP vs. the slope of the rapid phase of RVR response to a step increase in renal perfusion pressure of wild type (RGS2+/+) and RGS2 knockout (RGS2-/-) mice. Each point location represents the intersection of the RVR slope and baseline MAP of individual mice in each group. Values are mean ± SE. BW, body weight.

To determine the mechanism whereby loss of RGS2 results in decreased responsiveness of renal autoregulation, we assessed the kinetics of RBF, RVR and renal conductance in response to a step increase in renal perfusion pressure. The time course of changes in MAP, RBF, RVR, and renal conductance within the first 60 sec of superior mesenteric and celiac artery occlusion are shown in Fig 4. The initial sharp rise in MAP was similar between the two genotypes (Fig 4A). In both groups, RBF showed a biphasic response with initial increase prior to the subsequent decline to steady state levels below baseline within ~20 sec (Fig 4B). RVR rose rapidly within the first 20 sec following the step increase in MAP, and it continued to rise albeit at a slower rate until it plateaued at ~40 sec in both groups (Fig 4C). Changes in renal conductance followed a similar pattern but in the opposite direction as RVR (Fig 4D); however, the slower phase of decline in conductance in wild type was more apparent than in RGS2-/- mice. The rapid rise in RVR is similar to previous studies in which this phase of the autoregulatory response reflects the speed and/or strength of the myogenic response of the renal vascular bed[40, 41]. To determine the effect of RGS2 deficiency on the speed of myogenic response to increased renal perfusion pressure, we calculated the speed of the myogenic mechanism as the slope of a trendline drawn through 10 data points that spanned the first 10 seconds of the rapid rise phase of the RVR curve. As shown in Fig 4E, the slope of the RGS2-/- rapid phase trended lower relative to wild type slope (0.04 ± 0.01 vs. 0.05 ± 0.01 mmHg/μl/g body weight/s, *P* = 0.07). Because baseline MAP was different between the two groups, we next determined whether slower myogenic response in RGS2-/- mice was due to higher baseline renal perfusion pressure. Fig 4F shows that there was no correlation between baseline MAP and RVR slope. Altogether, these results suggest that RGS2 deficiency alters renal autoregulation by causing a slight reduction in the speed of the myogenic response.

### RGS2 deficiency causes a rightward shift of renal pressure-natriuresis relationship

**Fig 5:**
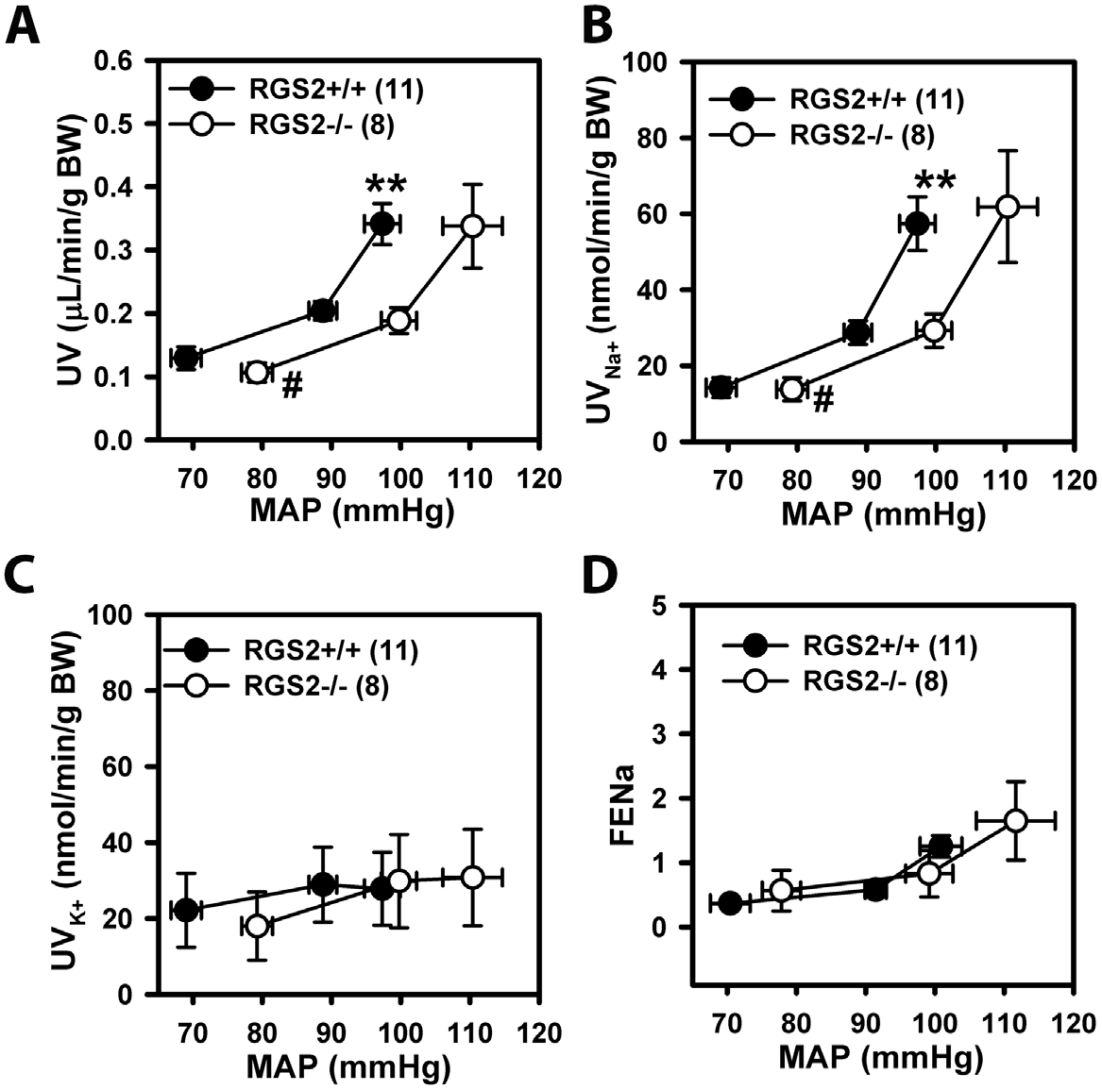
Effect of increasing renal perfusion pressure (MAP) on urine flow rate (A, UV), sodium excretion rate (UV_Na+_, B), potassium excretion rate (C), and fractional sodium excretion (D, FENa) in wild type (RGS2+/+, n = 12) and RGS2 knockout (RGS2-/-, n = 8) mice. Values are mean ± S.E. ^#^ *P* < 0.05 vs. RGS2+/+ baseline MAP; **P < 0.01 vs. RGS2+/+ UV and UV _Na+_ at equivalent MAP of ~100 mmHg. BW, body weight.

To determine further how renal RGS2 deficiency may lead to increased blood pressure, we assessed renal pressure-natriuresis relationships. Fig 5 shows the effects of increasing renal perfusion pressure on urine flow rate (diuresis) and sodium excretion (natriuresis) in uninephrectomized wild type and RGS2-/- mice under isoflurane anesthesia. Renal perfusion pressure increased ~20 mmHg in each genotype when the mesenteric and celiac arteries were occluded. Renal perfusion pressure increased further in both groups following occlusion of the abdominal aorta below the left kidney, in addition to occlusion of the mesenteric and celiac arteries. Renal perfusion pressure in wild type mice rose to 97 mmHg while RGS2-/- mice pressure rose to 110 mmHg. Under this condition, renal perfusion pressure (97 ± 3 mmHg) in wild type mice was equivalent to that of RGS2-/- mice prior to aortic occlusion (100 ± 2 mmHg). Thus, these conditions were used to compare the regulation of urine output, natriuresis, and renal hemodynamics by renal perfusion pressure in wild type and RGS2-/- mice at equivalent blood pressures. This analysis indicated that pressure-diuresis and pressure-natriuresis curves of RGS2-/- mice shifted to the right compared to wild type control mice (Fig 5A and 5B) with no apparent difference in the slope of the two curves (WT: 1.4 ± 0.6 vs. RGS2-/-: 1.4 ± 0.5 nmol/min/mmHg). At a renal perfusion pressure of ~100 mmHg, urine flow (WT: 0.34 ± 0.03 vs. RGS2-/-: 0.19 ± 0.02 μl/min/g body weight) and sodium excretion (WT: 57.3 ± 7.2 vs. RGS2-/-: 29.4 ± 4.1 nmol/min/g body weight) rates were reduced in RGS2-/- mice. In contrast, potassium excretion rates were similar between the two genotypes (WT: 27.9 ± 9.3 vs. RGS2-/-: 30.0 ± 12.3 nmol/min/g body weight). Together, these results suggested that in the absence of RGS2, there is a re-setting of the equilibrium pressure or the set-point at which sodium excretion matches sodium intake.

### Urinary sodium excretion following plasma volume expansion is decreased in RGS2-/- mice

**Fig 6:**
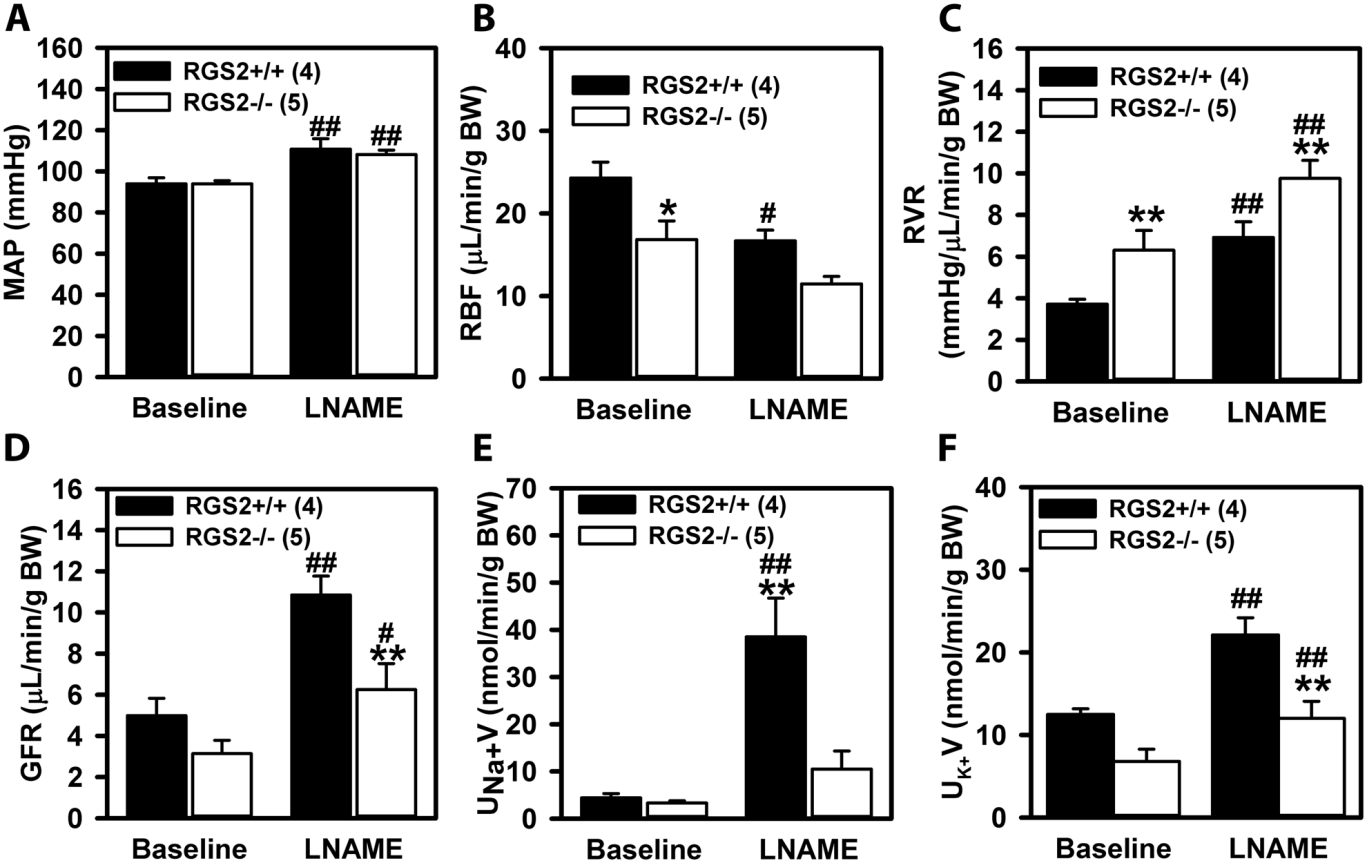
Effects of systemic administration of the non-selective eNOS inhibitor, L-NAME, on renal perfusion pressure (MAP), renal blood flow (RBF), renal vascular resistance (RVR), glomerular filtration rate (GFR), and sodium (UV_Na+_) and potassium (U_K+_V) excretion rate in wild type (RGS2+/+, n = 4) and RGS2 knockout (RGS2-/-, n = 5) mice. Values are mean ± SE. ^#, ##^ *P* < 0.05, 0.01 vs. corresponding baseline; *^,^**P < 0.05, 0.01 vs. RGS2+/+ mice. BW, body weight.

**Fig 7:**
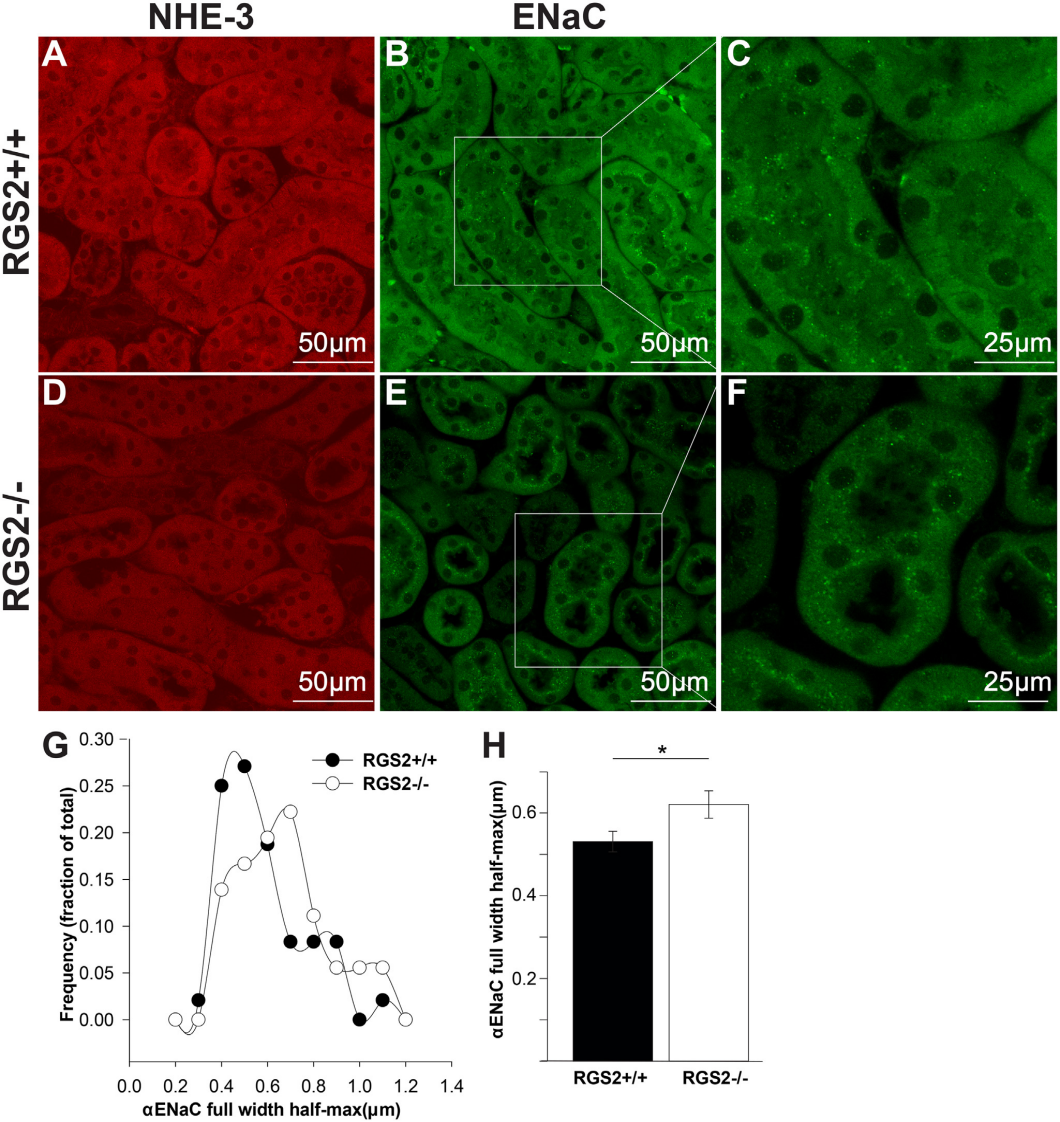
Analysis of renal tubular expression and distribution of sodium/proton exchanger-3 (NHE-3) and epithelial sodium channel (α-ENaC). A and D show representative images of expression and distribution of NHE-3 in wild type (RGS2+/+) and RGS2 knockout (RGS2-/-) renal tubules. B and E show representative images of α-ENaC expression and distribution. C and F are expanded inserts in B and E, respectively, showing a marked increase in luminal border localization of α-ENaC in RGS2-/- relative to wild type tubules. G, Frequency distribution of α-ENaC punctate size in the luminal border of renal tubules. H, average size of luminal α-ENaC granules. Values are mean ± SE. *P < 0.05 RGS2+/+ vs. RGS2-/- mice.

To determine whether the absence of RGS2 alters tubular sodium reabsorption, wild type and RGS2-/- mice were subjected to acute plasma volume expansion by continuous infusion of isotonic saline. To stimulate the excretion of excess water and sodium, arterial blood pressure was increased by systemic administration of the non-selective nitric oxide synthase inhibitor, L-NAME after baseline recordings. As expected, blood pressure, RVR and GFR increased whereas RBF decreased in both genotypes following L-NAME administration (Fig 6A–6D). Although sodium and potassium excretion rate increased in both genotypes following L-NAME injection, the natriuretic response was much more robust and reached significance only in wild type mice (Fig 6E and 6F), suggesting that sodium reabsorption was increased in RGS2-/- mice. To test this hypothesis, we determined whether RGS2 deficiency affected the expression and/or tissue distribution of Na^+^ channels in renal tubules. Tissue distribution (Fig 7A and 7D) and expression level of the proximal tubule sodium transporter, NHE-3, were unaffected by the absence of RGS2. In contrast, tubules from RGS2-/- mice showed increased luminal localization of ENaC, the distal tubule sodium transporter, whereas it was uniformly distributed in tubules from wild type animals (Fig 7B, 7C, 7E and 7F). Because ENaC can assemble and/or cluster in different stoichiometries and modular arrangements that can alter channel activity[44–48], we determined whether the absence of RGS2 affected the apparent size of luminal ENaC assemblies in renal tubules. As shown in Fig 7G, there was a heterogeneous population of luminal ENaC in the tubules of both genotypes. However, the average size of punctae of the highest frequency, determined from the frequency distribution plot (Fig 7G), was larger in RGS2-/- relative to those in wild type tubules (Fig 7H), suggesting a higher order arrangement or clustering in the absence of RGS2. These results together indicated that loss of RGS2 causes alterations in cellular distribution or assembly of ENaC that may promote sodium retention by the renal tubular system.

## Discussion

The absence of RGS2 in the kidney plays a causal role in the development of hypertension in RGS2-/- mice. However, the renal mechanisms that are affected in these mice are poorly understood. In this study, we have shown that RGS2 deficiency impairs renal function by augmenting renal vascular resistance, decreasing renal blood flow and glomerular filtration shifting the pressure-natriuresis relationship to higher pressures, and decreasing renal sodium excretion. These findings significantly expand understanding of how defects in renal mechanisms of blood pressure control contribute to hypertension due to the loss of *Rgs2*, a hypertension susceptibility gene. While renal dysfunction is a hallmark of essential hypertension, what still remain unresolved are the primary mechanisms and how they contribute to long-term blood pressure elevation.

Prior evidence and findings presented herein are consistent with the hypothesis that primary renal microvascular defects play a causal role in the development of hypertension in the absence of RGS2. We have found that elevated blood pressure in the absence of RGS2 is accompanied by increased RVR and impaired renal blood flow and GFR. These findings agree with those in previous work showing that 1) RGS2 tightly regulates signaling by G_q/11_ class G proteins[21, 22]; 2) several agonists including angiotensin II, endothelin-1, norepinephrine, and vasopressin that regulate renal function activate GPCRs coupled to G_q/11_[49, 50]; 3) signaling by G_q/11_ class G proteins plays key roles in several GPCR agonist-evoked physiological responses that are key to proper renal function, including vessel tone[51, 52]; and 4) renal interlobar arteries from RGS2-deficient mice show augmented vasoconstriction triggered by GPCRs coupled to G_q/11_ class G proteins[27]. Consistent with these lines of evidence, we found that renal blood flow at baseline is markedly decreased in RGS2-/- mice. Moreover, we found that basal renal vascular resistance is augmented in the absence of RGS2. Whereas renal autoregulation was responsive to a step increase in renal perfusion pressure in wild type and RGS2-/- mice, the sensitivity of the autoregulatory response was decreased in RGS2-/- mice, suggesting altered renal autoregulation in the absence of RGS2. We noted that the speed of myogenic mechanism of autoregulation in RGS2-/- mice tended to be slower in response to a step increase in renal perfusion pressure. This result was unexpected and at variance with the findings by Hercule et al[27]. In their study, interlobar arteries of RGS2-/- mice showed augmented myogenic response to increasing intraluminal pressure *ex vivo*, suggesting that pre-glomerular autoregulation is enhanced in RGS2-/- kidneys to limit the transmission of higher systemic pressure to the glomerulus. However, because RBF and GFR were attenuated in RGS2-/- mice, these changes in renal autoregulation may be maladaptive and further exacerbate or help perpetuate hypertension. In line with this suggestion, RGS2-/- renal microvessels have increased perivascular fibrosis that may occur as a consequence of renal hypo-perfusion and ischemic injury[53]. Without altering the magnitude of renal vascular myogenic response in RGS2-/- mice, renal perivascular fibrosis could also contribute to decreased sensitivity due to increased stiffness of the extracellular matrix of the vessel wall[54].

As previously reported, augmented vasoconstriction of RGS2-deficient arteries evoked by GPCR agonists may occur due to prolonged activity or exaggerated G_q/11_ signaling in the absence of RGS2[27]. In light of the additional findings in this study, what remain to be determined are the mechanisms by which regulation of G protein signaling by RGS2 modulates renal autoregulation in physiologically critical vessels, particularly pre- and post-glomerular arterioles. Achieving these goals would require a detailed assessment of the role of RGS2 in myogenic response and tubuloglomerular feedback, the principal mechanisms that mediate renal autoregulation.

*Does impaired RBF and GFR affect electrolyte and water homeostasis that ultimately impact long-term blood pressure homeostasis in RGS2 deficiency*? We have addressed this question by assessing the effect of RGS2 deficiency on the status of the pressure-natriuresis relationship. Our results show that the pressure-natriuresis curve of RGS2-/- mice is shifted rightward relative to wild type mice with no change in slope, indicating a re-setting of the equilibrium set-point to higher blood pressure. Although the shift in the acute pressure-natriuresis curve is similar to that observed in salt-independent hypertension[55], decreased sodium excretion rate in RGS2-/- mice at a renal perfusion pressure similar to wild type controls suggests that increased sodium retention contributes to the development of hypertension in the absence of RGS2. In agreement with this hypothesis, Gurley and colleagues previously showed that hypertension in RGS2 mice could be enhanced by high salt diet[30]. Furthermore, we observed increased luminal distribution or assembly of α-ENaC in RGS2-/- mice. The physiological relevance of this finding is that the insertion, retrieval or assembly of membrane ENaC subunits can vary in response to genetic or physiological perturbations. In this regard, kidneys from Dahl salt-sensitive rats, which transport twice as much sodium as their salt-resistant cohorts under baseline conditions[56], further increase membrane ENaC expression in response to high salt diet[47]. Furthermore, increased membrane localization of ENaC is associated with increased channel open probability and sodium transport in epithelial cells of distal nephrons[57, 58] and other organs[59, 60]. Thus, increased sodium retention due to increased tubular sodium reabsorptive capacity mediated by ENaC likely contributes to hypertension in RGS2-/-mice. Increased ENaC activity and sodium retention could also be facilitated by high order arrangement or clustering, favoring a particular stoichiometry of the subunits in the plasma membrane[48, 61]. In line with this suggestion, we found that luminal membrane localization and clustering of α-ENaC, were increased in RGS2-/- kidneys. However, what cannot be ascertained from the current study is whether alterations in tubular sodium handling in the absence of RGS2 occur as a consequence of a primary vascular defect causing decreased RBF and GFR, thereby re-setting the pressure-natriuresis relationship to achieve sodium balance, or whether higher perfusion pressure with lower GFR may be a consequence of primary perturbations in renal tubular sodium reabsorption mechanisms whereby increased sodium-retaining effects could be elicited by inappropriate activation of GPCR signaling in the absence of RGS2.

Our pressure-natriuresis experiments were performed under conditions in which kidney-extrinsic factors, i.e. neural and circulating hormonal input to the kidney, were clamped. While this protocol remains the optimal method to assess the role of hypertension candidate gene products in renal-intrinsic mechanisms as pertains to acute pressure-natriuresis relationship in mice under anesthesia, the use of continuous infusion of the hormone cocktail containing vasoactive agents may have obscured the effect of RGS2 deficiency on vascular-intrinsic mechanisms that influence renal function. Nonetheless, it is possible that RGS2 deficiency may have a direct effect on tubular sodium handling, since raising renal perfusion pressure in the absence of any hormone cocktail also led to decreased sodium excretion. We also note that glomerular/nephron number is ~30% higher in RGS2-/- compared to wild type controls despite a lack of difference in kidney weight. The overall glomerular surface area appears to be smaller; however, it remains to be determined whether such a difference between GFR and nephron number in RGS2-/- mice is part of a developmental adaptation elicited by persistent elevation of homeostatic blood pressure.

In conclusion, our study shows that RGS2 deficiency impairs renal function by decreasing glomerular filtration rate and renal blood flow, while augmenting renal vascular resistance and sodium retention. These findings may have implications in the choice of anti-hypertensive medication because, in hypertension patients who harbor *Rgs2* mutations that decrease protein expression and/or function, therapies that concurrently target vasoconstrictor and tubular GPCR signaling may be more effective drug of choice than those drugs that only affect one aspect of renal function.
